# Transcriptome Analysis of *Solanum Tuberosum* Genotype RH89-039-16 in Response to Chitosan

**DOI:** 10.3389/fpls.2020.01193

**Published:** 2020-08-05

**Authors:** Philipp Lemke, Bruno M. Moerschbacher, Ratna Singh

**Affiliations:** Institute for Biology and Biotechnology of Plants, University of Münster, Münster, Germany

**Keywords:** transcriptome analysis, RNAseq, potato, chitosan, defense, photosynthesis

## Abstract

Potato (*Solanum tuberosum* L.) is the worldwide most important nongrain crop after wheat, rice, and maize. The autotetraploidy of the modern commercial potato makes breeding of new resistant and high-yielding cultivars challenging due to complicated and time-consuming identification and selection processes of desired crop features. On the other hand, plant protection of existing cultivars using conventional synthetic pesticides is increasingly restricted due to safety issues for both consumers and the environment. Chitosan is known to display antimicrobial activity against a broad range of plant pathogens and shows the ability to trigger resistance in plants by elicitation of defense responses. As chitosan is a renewable, biodegradable and nontoxic compound, it is considered as a promising next-generation plant-protecting agent. However, the molecular and cellular modes of action of chitosan treatment are not yet understood. In this study, transcriptional changes in chitosan-treated potato leaves were investigated *via* RNA sequencing. Leaves treated with a well-defined chitosan polymer at low concentration were harvested 2 and 5 h after treatment and their expression profile was compared against water-treated control plants. We observed 32 differentially expressed genes (fold change ≥ 1; p-value ≤ 0.05) 2 h after treatment and 83 differentially expressed genes 5 h after treatment. Enrichment analysis mainly revealed gene modulation associated with electron transfer chains in chloroplasts and mitochondria, accompanied by the upregulation of only a very limited number of genes directly related to defense. As chitosan positively influences plant growth, yield, and resistance, we conclude that activation of electron transfer might result in the crosstalk of different organelles *via* redox signals to activate immune responses in preparation for pathogen attack, concomitantly resulting in a generally improved metabolic state, fostering plant growth and development. This conclusion is supported by the rapid and transient production of reactive oxygen species in a typical oxidative burst in the potato leaves upon chitosan treatment. This study furthers our knowledge on the mode of action of chitosan as a plant-protecting agent, as a prerequisite for improving its ability to replace or reduce the use of less environmentally friendly agro-chemicals.

## Introduction

Feeding an increasing world population remains one of the most important global tasks. In spite of the ongoing optimization of crops by modern plant breeding, agricultural production will be insufficient to nourish an expected ten billion people in 2050 unless agricultural practices are further improved. The situation is further exacerbated by a decline of farmland as a consequence of climate change and urbanization. With a global production of over 388 million tons in 2018 (FAOSTAT, www.fao.org), potato (*Solanum tuberosum* L.) is the world’s most important nongrain crop, exceeded only by the three top cereals, maize, wheat, and rice (Zaheer and Akhtar, 2016). Especially due to its high yield and nutritive composition including starch, vitamins, and antioxidants (Burlingame et al., 2009), potato is a crucial element in food security, particularly in developing countries of South America, Africa, and Asia (Scott and Suarez, 2012). Its autotetraploid and heterozygous nature, however, impedes the selection of desirable plant characteristics after crossing and is thus a challenge for modern breeding (Muthoni et al., 2015). To facilitate conventional breeding as well as genetic studies, diploid variants are used to overcome the complicated and difficult to trace heredity, and the homozygous, doubled-monoploid potato variant DM1-3 516 R44 was eventually used to sequence the potato genome in 2011 (Xu et al., 2011). This genome sequence was subsequently used to integrate data from the heterozygous diploid variant RH89-039-16, which more closely resembles commercial potato cultivars (Xu et al., 2011), hence opening new paths to investigate potato genomics and transcriptomics and thereby noticeably improving its breeding, cultivation, and protection.

In parallel to these efforts aiming to genetically improve crop cultivars, plant protection measures are essential to ensure crop yields and quality. These are not only subject to varying biotic and abiotic stress pressures, but also to changing legal regulations and consumer preferences. Increasing stress conditions due to climate change and increasing demand for healthy, residue-free, and sustainably produced crops, accompanied by a low acceptance of genetically engineered crop plants, call for the development of alternative or, rather, complementing plant production practices. Ideally, novel agro-chemical or agro-biological compounds should combine antimicrobial as well as plant-strengthening activities and simultaneously avoid environmental burdens. One of the most promising candidates for such an agrobiologic is chitosan. Chitosans are partially or fully *N*-deacetylated derivatives of chitin (poly-β-(1-4)-*N*-acetyl-D-glucosamine), the most abundant aminosugar biopolymer on earth. Chitin is naturally found, e.g., in the exoskeletons of arthropods and cell walls of fungi and yeast, acting as a structural polysaccharide (Rinaudo, 2006). Chitin is nontoxic, biodegradable, and an abundant renewable resource, but its crystallinity and, hence, insolubility severely limit its usability in agriculture. In contrast, chitosans, which are protonated and, hence, polycationic at slightly acidic pH values, are more easily soluble. Their solubility is strongly dependent on their degree of polymerization (DP) and, even more prominently, on their fraction of acetylation (FA), i.e., the mole fraction of anhydro-2-acetamido-2-deoxy-D-glucose units (Roberts, 2008). Furthermore, both DP and FA strongly affect the bioactivity of chitosans (Cord-Landwehr et al., 2020; Wattjes et al., 2020). Low FA chitosans have the highest antimicrobial activities (Omura et al., 2003; Gueddari and Moerschbacher, 2004; Younes et al., 2014), as reported against a broad range of plant pathogens, including oomycetes (Sharp, 2013), bacteria, fungi, viruses (Kulikov et al., 2006), and even herbivore insects (Rabea et al., 2005). On the other hand, intermediate to high FA chitosans seem to be best to elicit defense reactions in plants (Vander et al., 1998; Gueddari and Moerschbacher, 2004; Nietzel et al., 2018), e.g., triggering chitinase activity (Katiyar et al., 2014) as well as the biosynthesis of phenolics and phytoalexins (Bautista-Baños et al., 2006). While the antimicrobial activity of chitosans appears to depend on their polycationic nature favoring electrostatic interactions with negatively charged cell surfaces, their effect on plants is believed to be receptor mediated (Gueddari and Moerschbacher, 2004). In plants, chitosans most likely act as a pathogen or microbe associated molecular patterns (PAMP/MAMP) which are recognized through pattern recognition receptors (PRR) such as the chitin receptor CERK1 in *Arabidopsis* or CEBiP in rice (Iriti and Faoro, 2009). This so called PAMP-triggered immunity (PTI) is a well-studied and important defense system in plants which involves a number of signal transduction cascades, eventually resulting in the synthesis of proteins, reactive oxygen species (ROS), secondary metabolites, and phytohormones, to name just a few (Jones and Dangl, 2006; Miller et al., 2017).

In spite of the well-documented protective effects of chitosans in different plant species and against different pathogens (Hadrami et al., 2010), still surprisingly little evidence is provided concerning their actual mode of action, especially on a molecular level. Investigations regarding the effect of chitin oligomers on plants on a molecular level started in the early 2000s with microarray studies on *Arabidopsis* seedlings (Ramonell et al., 2002) and rice cell cultures (Akimoto-Tomiyama et al., 2003). Microarray studies were further pursued with chitosan oligomers on oilseed rape plants (Yin et al., 2006), *Arabidopsis* seedlings (Povero et al., 2011) and whole *Arabidopsis* plants (Jia et al., 2016). Only recently, first transcriptome studies using an RNAseq approach have been reported in chitosan-treated strawberry (Landi et al., 2017) and avocado (Xoca-Orozco et al., 2017) fruits. While a protective effect of chitosan treatment on potato, e.g., against *Phytophthora infestans* (O’Herlihy et al., 2003; Chang and Kim, 2012; Ippólito et al., 2017) or potato virus X (Chirkov et al., 2001) has been described, no such transcriptomic study has been conducted in potato yet. However, several transcriptome analyses on potato have been published, e.g., describing gene expression under drought stress (Gong et al., 2015) and other abiotic and biotic stresses (Massa et al., 2013), mostly based on the reference data for the potato transcriptome by Massa et al. (2011). Here, we report on a whole transcriptome analysis of potato genotype RH89-039-16 following treatment with a well-defined chitosan.

## Material and Methods

### Chitosan

The chitosan used in this study was derived from shrimp shell α-chitin and received from Mahtani Chitosan Pvt. Ltd. (Veraval, India). It had an average FA of 0.2 as determined *via* proton nuclear magnetic resonance (^1^H-NMR) (Hirai et al., 1991; Lavertu et al., 2003), and a weight average molecular weight (Mw) of 87 kDa (DPw 515), as determined using size exclusion chromatography coupled to refractive index detection and multiangle laser light scattering (HPSEC-RI-MALLS) (Schatz et al., 2003). The dispersity Đ of the DP was determined as Đ_DP_ = Mw/Mn = 2. Chitosan solutions were obtained by dispersing chitosan powder in distilled water and solubilization with a 5% molar excess of acetic acid relative to the free amino groups in the chitosan used.

### Potato Cultivation and Leaf Treatment

Potato plants obtained from tubers were cultivated in an environmental chamber with constant temperatures under long-day conditions (16/8 h photoperiod, 24/18°C). One day before treatment, mature leaves were detached from fully developed side shoots and collected on petri dishes containing water agar (0.05% agar in distilled water). The petri dishes were closed and incubated in the environmental chamber for 24 h. This procedure ensured the de-stressing of the leaves overnight after being detached from the plant. For treatment, the abaxial surfaces of the leaves were sprayed with a 0.05% chitosan solution, or with distilled water as a control, until small droplets formed on the leaf surface. Treated leaves were incubated in the environmental chamber again before being frozen in liquid nitrogen 2 and 5 h after treatment. These times were chosen as a result of extensive pretests and quantitative PCR (qPCR) studies using potentially chitosan-triggered genes including WRKY transcription factors and resistance genes, aimed to identify appropriate time points for the transcriptome analysis. This resulted in a total of twelve samples, with two time points, two treatments, and three biological replicates each.

### RNA Isolation

To isolate the RNA from the frozen leaves, leaf tissue was ground with mortar and pestle under liquid nitrogen. Extraction of RNA from the leaf powder was done using the innuPREP RNA Mini Kit from Analytik Jena (Jena, Germany). DNA was removed with a provided precolumn which eliminated DNAse digestion. RNA quality and quantity were determined *via* Nanodrop 2000 (Thermo Fisher Scientific, Waltham, Massachusetts, USA) and a Bioanalyzer 2100 (Agilent Technologies, Santa Clara, California, USA) prior to sequencing.

### RNA Sequencing

The sequencing library was prepared following the Illumina TruSeq mRNA protocol (https://support.illumina.com/downloads/truseq_stranded_mrna_sample_preparation_guide_15031047.html). The sequencing was conducted with an Illumina HiSeq 3000 (Illumina, San Diego, California, USA) with all 12 samples on one HiSeq lane, resulting in 21-26 million 50-bp-reads per sample. After quality control of the raw sequencing data, all reads were computationally aligned to the potato reference genome PGSC v4.03 (Xu et al., 2011). RNAseq quality control and sequencing procedures were performed by GeneVia Technologies (Tampere, Finland), as described below.

#### Data Analysis

The analysis was started from raw sequencing data in fastq format. The reference genome and its associated annotation gff file were obtained from a specific potato Ensembl page (ftp://ftp.ensemblgenomes.org/pub/release-46/plants/gtf/solanum_tuberosum).

#### Quality Control

Quality of the RNAseq reads was inspected using FastQC software (Frenkel, 2009). TrimGalore! (Krueger, 2018) was ran on the reads, with default settings.

#### Read Alignments and Read Counts

RNAseq reads were aligned to *Solanum tuberosum* reference genome Soltub 3.0 using STAR aligner, version 2.5.2 (Dobin et al., 2013). Gene-level read counts were obtained simultaneously with the alignment process. For visual exploration of the data, the obtained read counts were normalized using regularized log transformation function of DESeq2 R package (Love et al., 2014), which transforms the count data to the log_2_ scale in a way that minimizes differences between samples for rows with small counts and also normalizes the data with respect to library size. A visual inspection of the samples using principal component analysis (PCA) and a Pearson’s correlation heatmap was followed by combining the technical replicates of each sample by averaging their gene counts for subsequent analysis steps.

#### Differentially Expressed Genes

Data normalization and differential expression analysis were performed using R package DESeq2 (Love et al., 2014). The data was divided per comparison into four groups: CS_5h vs CS_2h, CS_2h vs H_2_O_2h, H_2_O_5h vs H_2_O_2h and CS_5h vs H_2_O_5h. For each of these, pairedness was included in the design matrices as shown in **Supplementary Table 1**. Heatmaps and volcano plots of the differentially expressed genes were created using pheatmap (Kolde, 2019) and an in-house volcano-visualization function, based on ggplot2 (Wickham, 2011), respectively. Furthermore, a heatmap only including genes upregulated both 2 and 5 h after treatment was created using the iDEP (integrated Differential Expression and Pathway analysis) webtool (http://bioinformatics.sdstate.edu/idep/, accessed on 07.07.2020), comparing gene expression between both times using mean-centered fold change values (Ge et al., 2018). Genes having adjusted p-value <0.05 and absolute log_2_ fold change >1 were considered significantly differentially expressed.

#### Enrichment Analysis

The above groups of differentially expressed genes (DEGs) from each comparison were subjected to enrichment analysis of associations to Gene Ontology (GO) biological process terms. The enrichment analyses determined whether any GO terms are annotated to a list of specified genes, in this case a list of DEGs, at a frequency greater than what would be expected by chance and calculated a p-value using hypergeometric distribution. A file containing transcript IDs and corresponding protein IDs [http://rsat.eead.csic.es/plants/data/genomes/Solanum_tuberosum.DM.v4.03.PGSC/genome/peptidic_sequences.fasta] was used to associate PGSC transcript IDs to Ensembl protein IDs. A table [http://bioinfo.cau.edu.cn/agriGO/download/item2term_73], displaying correspondence between Ensembl protein IDs and GO entries was used to associate the proteins to GO entries. Finally, the GO terms were associated with their corresponding descriptions using the R GO database (Meurk et al., 2013) and deprecated terms were removed. The conversions returned altogether 13,656 genes with GO term associations. All potato genes with GO annotation were used as background set for the enrichment analysis. The p-values of enrichment analysis were corrected for multiple testing using Benjamini-Hochberg multiple testing adjustment procedure (Hochberg, 1995). GO terms with adjusted p < 0.05 and with at least two genes from a gene group studied were considered significantly enriched. Enrichment analysis was further broadened conducting a cluster-wise enrichment analysis *via* iDEP (integrated Differential Expression and Pathway analysis) webtool (http://bioinformatics.sdstate.edu/idep/, accessed on 07.07.2020) (Ge et al., 2018), including all available gene sets for pathway analysis.

#### MapMan and Kyoto Encyclopedia of Genes and Genomes Pathway Annotation

MapMan software was used to display the DEG dataset in the context of biological functions and pathways (Thimm et al., 2004; Usadel et al., 2009). To insert the gene expression data, the “Stub_PGSC_DM_v3.4” mapping file for the *S. tuberosum* genome was used which is accessible on the MapMan website. Likewise, the Kyoto Encyclopedia of Genes and Genomes (KEGG) database was used to visualize the DEG dataset and to further illustrate the gene functions (Ogata et al., 1999). The usage of corresponding KEGG pathways were officially granted prior to publication (Kanehisa et al., 2017; Kanehisa et al., 2019).

### Validation of DEGs *via* Real-Time qPCR

To validate the gene expression data from the RNAseq, five representative photosynthesis-related DEGs were selected for qPCR studies. qPCR runs were carried out in three independent experiments with triplicates of all samples in each experiment. To minimize natural variation that occurs when using different plants, particularly plants cultivated in different seasons, the same RNA samples were used for qPCR quantification and RNAseq analysis. After extracting the RNA as described above, first strand cDNAs were synthesized from 500 ng of total RNA using PrimeScript RT Master Mix from Takara Bio Inc. (Kusatsu, Shiga, Japan), following the manufacturer’s instructions. To conform to qPCR standards, highly specific primers were designed using NCBI Primer Blast (Ye et al., 2012) with melting temperatures (Tm) between 59 and 61°C, 20 bp length and amplicon lengths of 100-200 bp. Self- and cross dimerization of primers was excluded by running the Multiple Primer Analyzer webtool from Thermo Fisher Scientific (Waltham, Massachusetts, USA). As references, housekeeping genes of the elongation factor 1-α (ef1α) and the 18S rRNA were used as previously used in qPCR and RNAseq studies with potato plants (Nicot et al., 2005; Goyer et al., 2015). The primers for the reference genes were designed and approved likewise. All primers are listed in **Supplementary Table 2**. Nontemplate controls were included for each primer pair to exclude false-positive results due to unspecific dye binding. The qPCR cycler was a CFX96 Touch Real-Time PCR Detection System (Bio-Rad Laboratories, Inc., Hercules, California, USA), with initial denaturation at 95°C for 3 min followed by 44 cycles of 95°C for 3 s and 60°C for 20 s. Melting curve analysis was performed from 58 to 95°C, where the temperature increased by 0.5°C every 5 s. The total volume was 10 µl per sample, containing 2.5 µl of cDNA (1:50 dilution from cDNA synthesis samples, i.e., 50 ng), 2.5 µl of a mix of one primer pair (1:250 dilution from 100 µM stock solutions, i.e., 0,4 µM per primer) and 5 µl of KAPA SYBR FAST qPCR Master Mix (Sigma-Aldrich, St. Louis, Missouri, USA). Primer efficiencies were determined using a high-quality cDNA template (as determined *via* Nanodrop) in several factor 10 dilution steps (1:10 to 1:1,000,000), resulting in a standard durve by plotting the log of the cDNA quantity against the cycle threshold value obtained during amplification. An R^2^ value > 0.9 was considered as sufficient fitting of the experimental data to the regression line. The primer or amplification efficiency E was eventually calculated from the standard curve slope by the formula E = 10^-1/slope^. Primer efficiencies are given in **Supplementary Table 2**. Analysis of qPCR data was done using the efficiency corrected calculation model described by (Pfaffl, 2001). REST-MCS (relative expression software tool – multiple condition solver) was used to allow the direct comparison of both time points and both treatments in one analysis (Pfaffl, 2002).

### Oxidative Burst Assay

To investigate the eliciting activity of chitosan on potato leaves, leaf disks were prepared from fully-grown, mature RH89-039-16 potato leaves that were freshly detached from the plant. Disks were cut *via* gently pressing with a cork borer (Ø 5 mm) on the lamina part of the leaf, avoiding strong veins and the midrib. Each disk was subsequently transferred to a well of a 96 well microtiter plate containing 100 μl of dH2O. The plates were covered with aluminum foil and incubated at room temperature over night to prevent interference of ROS produced after wounding by allowing de-stressing of the freshly cut leaf disks (Bredow et al., 2019). After overnight incubation, the water was replaced by 200 μl of 0.05% chitosan and 0.5 mM of the luminol derivative L-012 (8-amino-5-chloro-7-phenylpyrido[3,4-d]pyridazine- 1,4(2H,3H)dione) (Nishinaka et al., 1993) in 10 mM MOPS/KOH buffer (pH 7.4). H_2_O_2_ was quantified by a microplate reader measuring the light emission caused by the reaction of H_2_O_2_ and L-012 (Albert and Fürst, 2017). Chemiluminescence was continuously measured for 5 s per well over a total time of 90 min and is given as relative light units (RLU).

## Results

### RNAseq Data Analysis

#### Sample Quality Control and Read Alignment

Total RNA quality tested by Bioanalyzer displayed RIN values ≥8 and 25/18 s ratios between 1.9 and 2.5 for all samples. The quality of all processed samples was also found to be good and consistent, only displaying a slight TruSeq adapter contamination, that was taken care of by a run of TrimGalore!, using default parameters. The alignment statistics are presented in **Supplementary Table 1**. Uniquely mapped alignment rates were consistently above 80% for all samples, except for the CS_5h samples which had >25% of multimapped reads, possibly indicating ribosomal RNA (rRNA) contamination. However, the read counts for differential expression analysis were calculated using only the uniquely mapped reads, and since the total number of reads was high in all samples, this was concluded not to cause problems in differential expression analysis.

#### Principal Component Analysis and Pearson’s Correlation Heatmap

Both PCA and Pearson’s correlation coefficient calculations were performed as final methods to ensure data quality. In the PCA including all samples, the two first principal components explained 70.2% of the variance between samples. According to both the visualization of the PCA and Pearson’s correlation analysis, the samples did not cluster clearly together by treatment. This may be due to high sample similarity as indicated by the Pearson’s correlation values (**Supplementary Figure 1**). **Supplementary Figure 2** presents the visualization of PCA results on samples of one time point but different treatment, which all in all showed that the samples still showed a certain grouping by treatment.

### Differentially Expressed Genes

**Figure 1:**
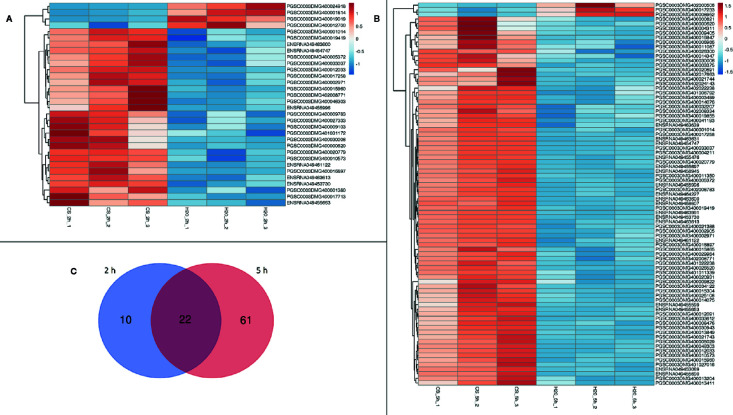
Differentially expressed genes in a cluster-wise heatmap of all biological replicates 2 h after chitosan treatment **(A)**, 5 h after chitosan treatment **(B)** and in a Venn diagram visualizing time point overlapping gene expression **(C)**. The data for the heatmap is log_2_ transformed and clustered by rows and columns. The Venn diagram shows separate gene expression for the first time point (2 h) in blue and separate gene expression for the second time point (5 h) in red.

Two hours after treatment, the analysis of differential gene expression yielded a total of 32 DEGs for the comparison chitosan-treated versus water-treated leaves (**Figure 1A**), while 5 h after treatment 83 DEGs were found (**Figure 1B**). Gene expression was clearly in chitosan-treated samples 5 h after treatment in comparison to 2 h after treatment (**Supplementary Figure 3**). **Figure 1** shows the clustering of the DEGs based on their similar expression patterns. In both sample groups (chitosan versus water 2 h after treatment and chitosan versus water 5 h after treatment), the smaller cluster represents the downregulated DEGs while the larger one represents the upregulated DEGs. Consequently, 2 h after chitosan treatment, 28 genes were detected to be upregulated and 4 genes were found to be downregulated, whereas 5 h after chitosan treatment three genes were downregulated and 80 were upregulated. Of all DEGs, 10 were exclusively differentially expressed 2 h after chitosan treatment, while 61 DEGs were exclusively differentially expressed 5 h after chitosan treatment. Twenty-two of all upregulated DEGs were differentially expressed at both time points (**Figure 1C**).

Genes of unknown function are not discussed further as no clear allocation to certain functions or targets are possible. Interestingly, we observed that chitosan treatment mostly triggered the expression of genes related to electron transport in mitochondria and chloroplasts, including both nuclear- and chloroplast-encoded genes. **Supplementary Table 3** lists all genes mentioned in the following chapters, including their full description and transcriptomic gene IDs. The full lists of differentially expressed genes including their fold changes and p-values are reported in the **raw data** provided.

#### Chitosan-Triggered Downregulation of Genes Was Limited to Very Few Genes

Functional annotation of downregulated genes showed that they include structural constituents of ribosomes and genes associated with peptide metabolic processes. Another downregulated DEG was identified as a CoA hydrolase and was observed enriched in the KEGG ribosome pathway. Furthermore, an ethylene-responsive transcription factor, identified enriched in a cellular defense response pathway and Alpha-DOX2, enriched in KEGG alpha-linolenic acid metabolism, were downregulated 5 h after chitosan treatment. However, none of the downregulated genes showed a similar expression pattern in both sample groups.

#### Chitosan Treatment Induces Few Genes Directly Involved in Disease Resistance

**Figure 2:**
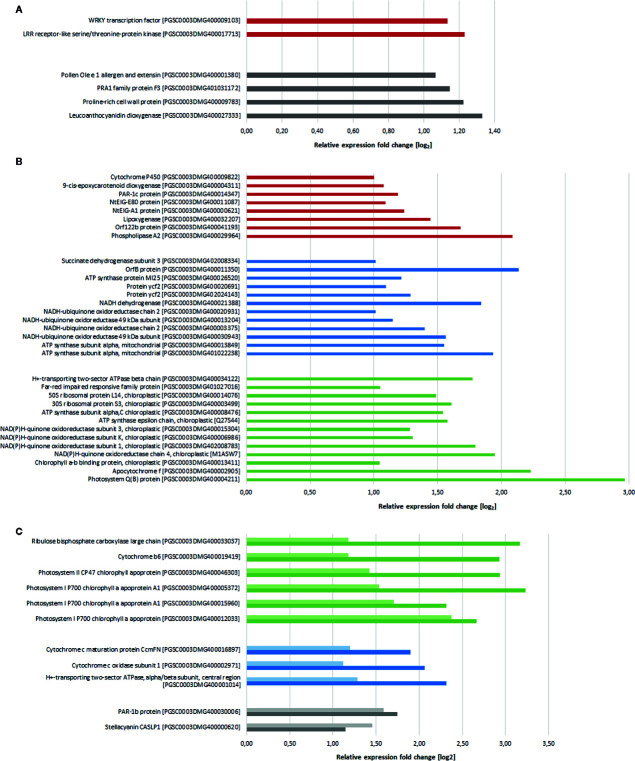
Differentially expressed genes (log_2_ fold change > 1, p-value < 0.05) after chitosan treatment in comparison to water treatment. **(A)** Gene expression 2 h after treatment. **(B)** Gene expression 5 h after treatment. **(C)** Genes differentially expressed at both 2 h (light colored) and 5 h (dark colored) after treatment. Red bars show defense-related genes, blue bars show basic metabolism related genes, green bars show photosynthesis-related genes, genes with other functions are grouped and indicated with gray bars. Genes of unknown function as well as genes coding for ribosomal proteins are not shown.

Although activation of plant defense is widely known as one of the main reactions of plants to chitosan, we observed expression of only few defense-related genes. Potentially defense-related genes that were upregulated 2 h after chitosan treatment coded for a leucine-rich repeat receptor like kinase (LRR-RLK) and a WRKY transcription factor (**Figure 2A**). In addition, genes coding for an extensin and a proline-rich cell wall protein, both involved in cell wall synthesis and alteration and hence with potential to participate in defense responses, were upregulated. Several defense- and stress-related genes were in addition upregulated 5 h after treatment, including genes coding for photoassimilate-responding proteins, as well as for proteins to withstand osmotic, salt, and drought stress (**Figure 2B**). This surprisingly low number of upregulated defense-associated genes may indicate that chitosan is inducing plant disease resistance in a rather indirect manner, by triggering alterations in gene expression predominantly targeting cellular functions not solely or directly involved in pathogen defense.

#### Chitosan Treatment Activates Mitochondrial Gene Expression

One major consequence of chitosan treatment was the activation of (nuclear) genes coding for mitochondrial proteins. For example, genes encoding for cytochrome c oxidase (mitochondrial complex IV) and ATP synthase subunits were upregulated already 2 h after chitosan treatment with further increasing expression at the later time point (**Figure 2C**). Especially 5 h after chitosan treatment several genes related to mitochondrial electron transport (and hence, cellular energy supply) were upregulated, e.g., further ATP synthase subunits and genes related to mitochondrial complexes I and II.

**Figure 3:**
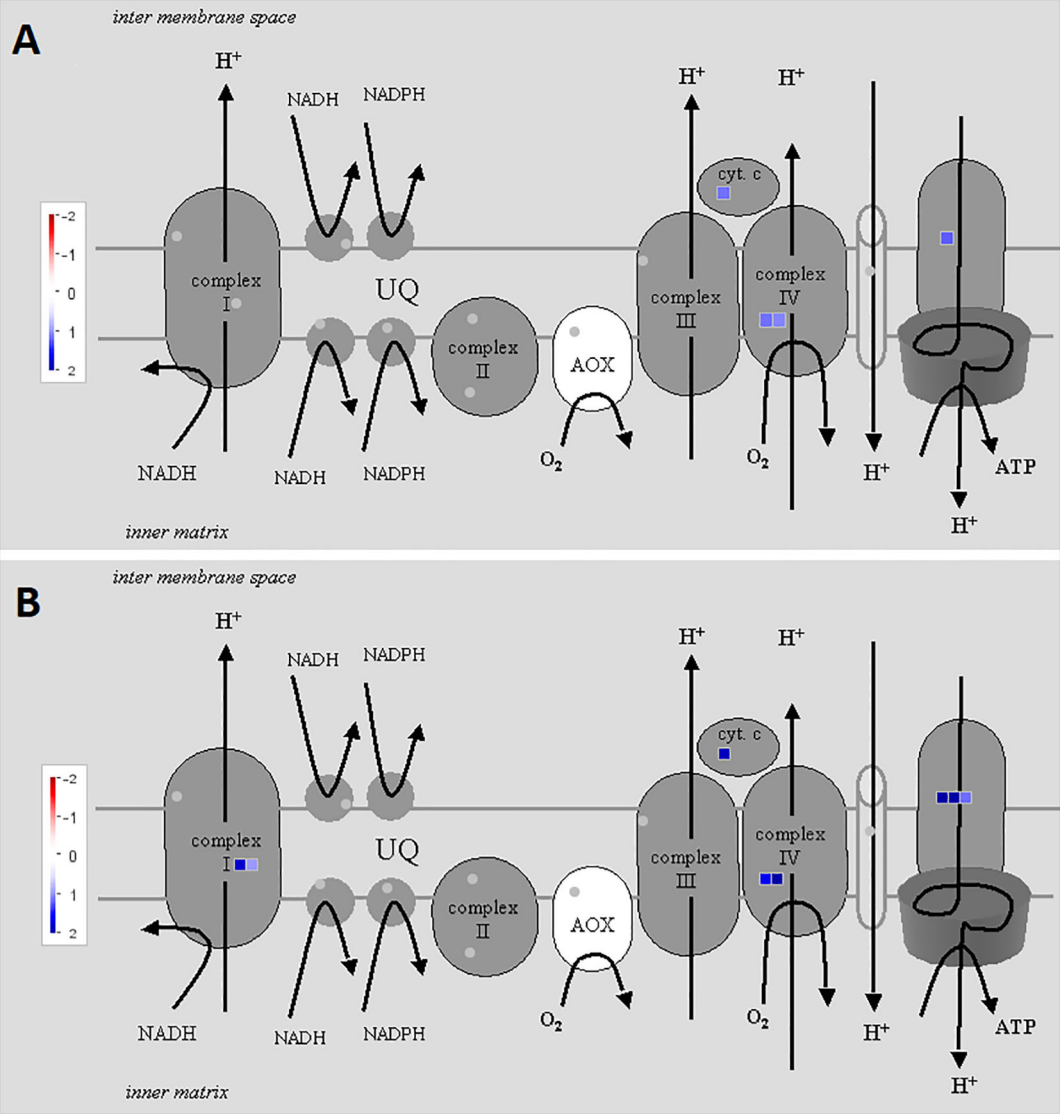
Differentially expressed genes (DEGs) related to mitochondrial electron transport at time point 2 h **(A)** and 5 h **(B)**. The fold change was analyzed and allocated with MapMan software. Blue squares show the intensity of upregulation, where darker blue color indicates stronger upregulation. To visualize the pathway, Mitochondrial_e-transport 5.01 pathway was used. Stub_PGSC_DM_v3.4 was utilized for mapping as this mapping file represents the reference genome used for the sequencing. Both pathway and mapping were obtained from the MapMan website and are available through the Creative Common (CC) license. The mapping file did not cover all complex I and complex II related genes.

**Figure 4:**
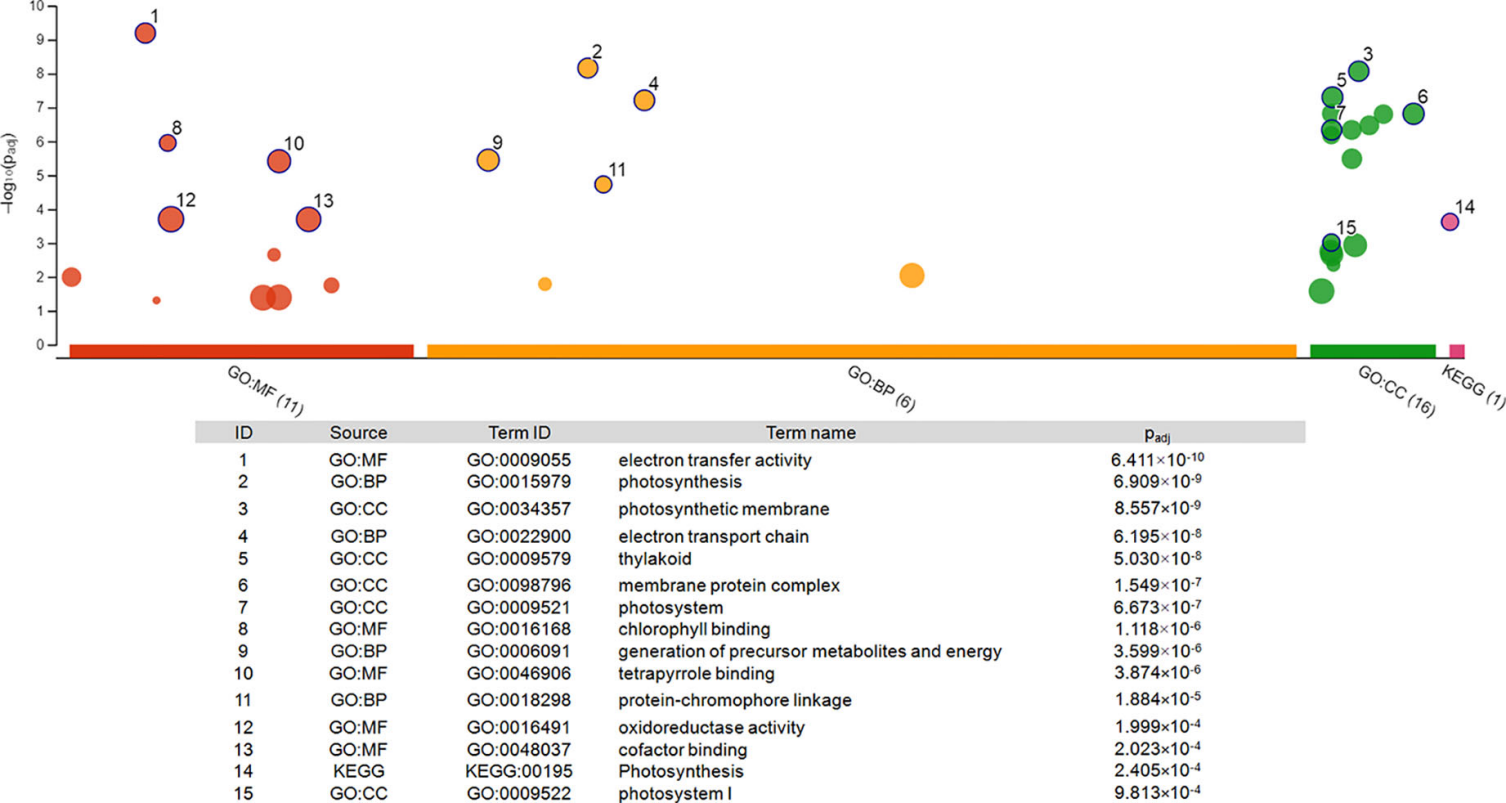
Pathway enrichment analysis of the 22 genes differentially expressed at both time points. The graph was generated *via* gProfiler service g:GOSt. X-axis represents grouped and color-coded functional GO terms (with numbers indicating the amount of significantly enriched terms in the corresponding source), y-axis shows the adjusted enrichment p-values in negative log_10_ scale. Dot size in the picture indicates the abundance of enriched genes in the functional groups. Numbers displayed on the dots are related to the details given in the table below the picture.

To illustrate the activation of electron transport in mitochondria, MapMan software with the “Mitochondrial_e-transport” pathway file was used (**Figure 3**). Corresponding genes were triggered both 2 and 5 h after chitosan treatment, indicated by upregulation of genes coding for subunits of complex I, cytochrome c protein, complex IV, and ATP synthase and further proven by a high enrichment of electron transfer activity on the molecular function (MF) level (**Figure 4**). As mentioned before, gene expression was stronger 5 h after chitosan treatment, and induction of genes coding for complex I was observed exclusively at this time point. Furthermore, ATP synthase gene expression was more strongly upregulated 5 h after chitosan treatment.

The upregulation of genes related to electron transport in mitochondria, especially with the increasing fold change over time, provides strong evidence for chitosan triggering mitochondrial cell activity as one of its main mechanism of action in the first hours after plant treatment.

#### Photosynthesis-Related Gene Induction Is the Main Response to Chitosan Treatment

While differential gene expression 2 h after chitosan treatment can be allocated to different biological functions, DEGs 5 h after treatment are mostly involved in photosynthesis and respiration (**Figures 2B, C**). For example, four different genes coding for subunits of the chloroplast NADH dehydrogenase-like (NDH) complex were upregulated. This was accompanied by upregulation of four genes coding for ATP synthase subunits. The highest upregulation of photosynthesis-related genes was displayed by genes coding for the PSII subunit D1 (*psbA*, 2.9-fold upregulation) and the cytochrome f subunit of the cytochrome b_6_f complex (*petA*, 2.2-fold upregulation). Furthermore, from 11 genes that are upregulated at both time points (**Figure 2C**), six can be clearly allocated to the light phase of photosynthesis, including genes coding for PSI and PSII subunits. Furthermore, cytochrome b_6_ subunit (*petB*) of the cytochrome b_6_f complex was upregulated at both time points. These genes all showed higher upregulation after 5 h than after 2 h.

**Figure 5:**
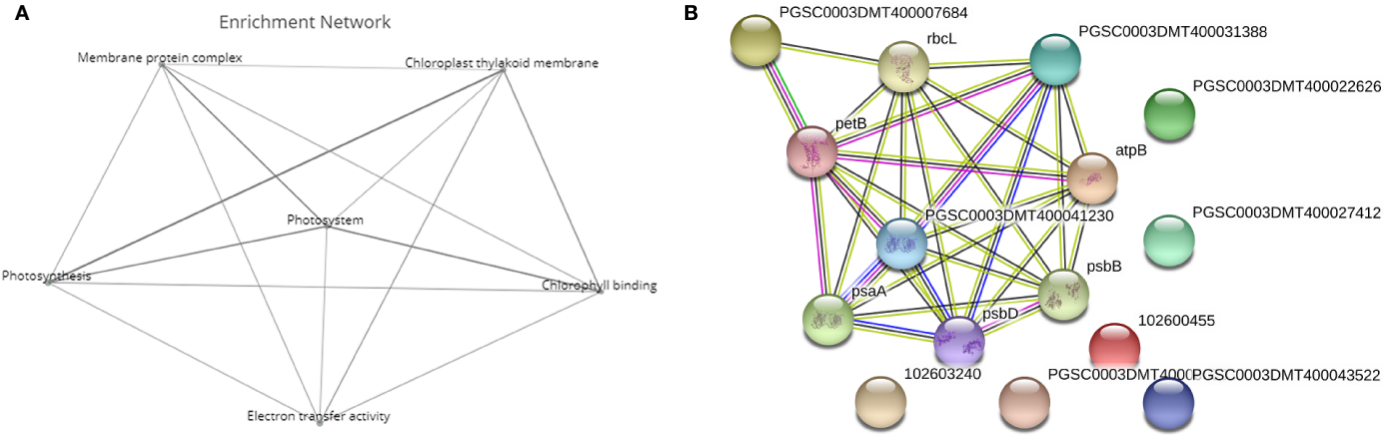
Deeper analysis of pathway enrichment. **(A)** Enrichment network among the functional pathways. **(B)** Protein-protein interaction network generated *via* Search Tool for the Retrieval of Interacting Genes/Proteins (STRING) displaying the interaction among the genes in the enriched pathways. Four different evidence types were used to predict protein associations: black lines indicate coexpression evidence, green lines indicate neighborhood evidence, purple lines indicate experimental evidence and blue lines indicate cooccurrence evidence.

Functional enrichment of the 22 DEGs which showed upregulation at both 2 h and 5 h after chitosan treatment with iDEP demonstrated that upregulated DEGs were significantly enriched in six, mainly photosynthesis-related, pathways (**Figure 5A**). Interestingly, the most upregulated (log_2_ fold change ≥ 3) DEGs were identified enriched in the electron transport pathways and photosynthesis, supporting our GO enrichment analysis (**Figure 3**), which showed that DEGs are related to photosynthesis pathways on the biological function (BF) and the cellular component (CC) levels.

**Figure 6:**
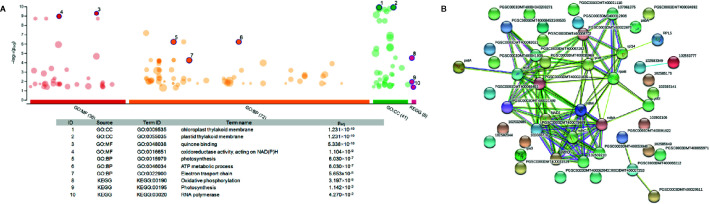
Enrichment analysis **(A)** and protein-protein interaction network **(B)** of the unique DEGs 5 h after chitosan treatment. **(A)** was generated *via* gProfiler service g:GOSt. X-axis represents grouped and color-coded functional Gene Ontology (GO) terms (with numbers indicating the amount of significantly enriched terms in the corresponding source), y-axis shows the adjusted enrichment p-values in negative log_10_ scale. Dot size in the picture indicates the abundance of enriched genes in the functional groups. Number displayed on the dots are related to the details given in the table below the picture. **(B)** Four different evidence types were used to predict protein associations: black lines indicate coexpression evidence, green lines indicate neighborhood evidence, purple lines indicate experimental evidence and blue line indicate cooccurrence evidence.

To further analyze the interaction among the DEGs enriched in the MF, BF and CC pathways, a protein-protein-interaction (PPI) network was built *via* Search Tool for the Retrieval of Interacting Genes/Proteins (STRING, **Figure 5B**). This network among the upregulated DEGs at both time points shed a light on the coexpression of the genes related to photosystems, electron transport and ATP synthesis and their connection in the enriched pathways. Upregulated and functionally annotated genes enriched in these pathways were psbA, psbB, and pdbD, coding for three essential subunits of photosystem II, petB, an important part of the cytochrome b_6_f complex as well as rbcL, a gene coding for a RuBisCo subunit. **Figure 6** shows the enrichment analysis **(A)** and PPI **(B)** of DEGs upregulated 5 h after chitosan treatment and the relation among the genes linked to photosynthesis, ATP metabolism and oxidoreductase activity. Outstanding DEGs again included psbA and petA, similar to the PPI analysis 2 h after chitosan treatment, but also ndh and ATP synthase genes, likewise acting in photosynthesis. Cluster-wise analysis of DEGs at both time points emphasized the hypothesis of chitosan-dependent photosynthesis activation as all annotated pathways were linked to the light reaction (**Table 1**).

**Table 1:** Enriched pathways for each cluster.

Cluster	Adjusted p-value	Number of genes	Pathways
A	7.5e04	2	Photosystem I
	1.9e-03	2	Electron transport chain
	9.6e-03	2	Tetrapyrrole binding
B	6.8e-09	5	Photosynthesis
	6.8e-09	5	Electron transfer activity
	3.5e-08	5	Membrane protein complex

Cluster-wise enrichment analysis was conducted using R-based iDEP (integrated Differential Expression and Pathway analysis) online tool.

Gene categories containing genes that are upregulated 2 h after chitosan treatment (**Supplementary Table 4**) were mainly thylakoid components (GO:00095793), PSI (GO:0009522), and general photosynthesis processes (GO:0015979 and GO:0019684). The same genes were upregulated 5 h after chitosan treatment (**Supplementary Table 5**).

**Figure 7:**
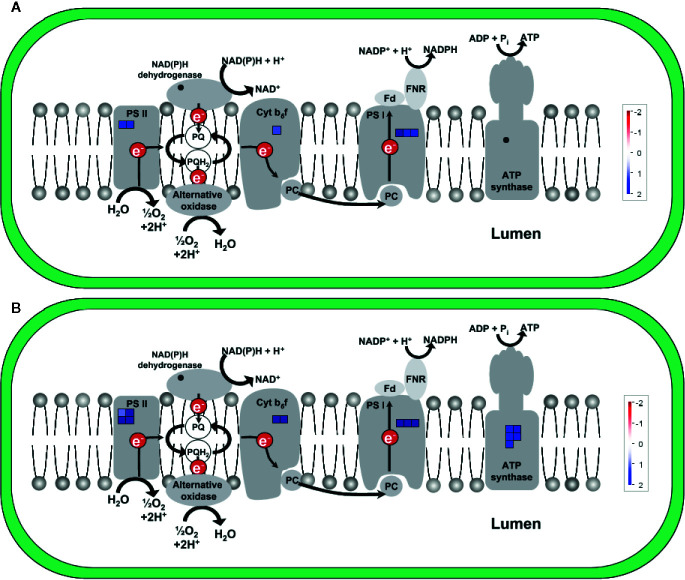
Differentially expressed genes (DEGs) related to electron transport in the light reaction of photosynthesis in the chloroplast at time point 2 h **(A)** and 5 h **(B)**. The fold change was analyzed and allocated with MapMan software. Blue squares show the intensity of upregulation, where darker blue color indicates stronger upregulation. To visualize the pathway, ChloroPlast_CustomArray_CUSTOM_MAPPING 1 pathway was used. Stub_PGSC_DM_v3.4 was utilized for mapping as this mapping file represents the reference genome used for the sequencing. Both pathway and mapping were obtained from the MapMan website and are available through the Creative Common (CC) license. NDH complex is not covered by the pathway.

**Figure 7** shows the upregulation of genes related to electron transport in the light reaction of photosynthesis in the chloroplast, visualized *via* MapMan (using the “ChloroPlast_CustomArray_CUSTOM_MAPPING” pathway file). Genes coding for subunits of PSII, cytochrome b_6_f complex, and PSI were upregulated at both time points and upregulation was clearly stronger 5 h after chitosan treatment, including the upregulation of more genes for PSI subunits. In addition, D1, one of the two large subunits of PSII, was only upregulated 5 h after chitosan treatment. The largest difference between the two time points concerning chloroplast activity is the upregulation of five genes coding for ATP synthase subunits exclusively at 5 h after chitosan treatment (**Figure 7B**).

To further visualize the activation of photosynthesis-related genes, assignment of upregulated genes to their respective structure in the photosynthetic apparatus was also done *via* KEGG pathway map for the photosynthetic light reaction in potato (Ogata et al., 1999). The KEGG pathway map indicates the activation of genes coding for subunits of all four main light reaction components, namely PSII, cytochrome b_6_f complex, PSI, and ATP synthase (**Supplementary Figure 4**), all of them encoded in the chloroplast (Rogalski et al., 2015).

The significant upregulation of photosynthesis-related genes, mainly of the light reaction in chloroplasts, provides strong evidence that chitosan treatment is predominantly triggering increased electron transport in chloroplasts, eventually leading to higher concentrations of sugars and energy itself.

### Validation of DEGs *via* qPCR

**Figure 8:**
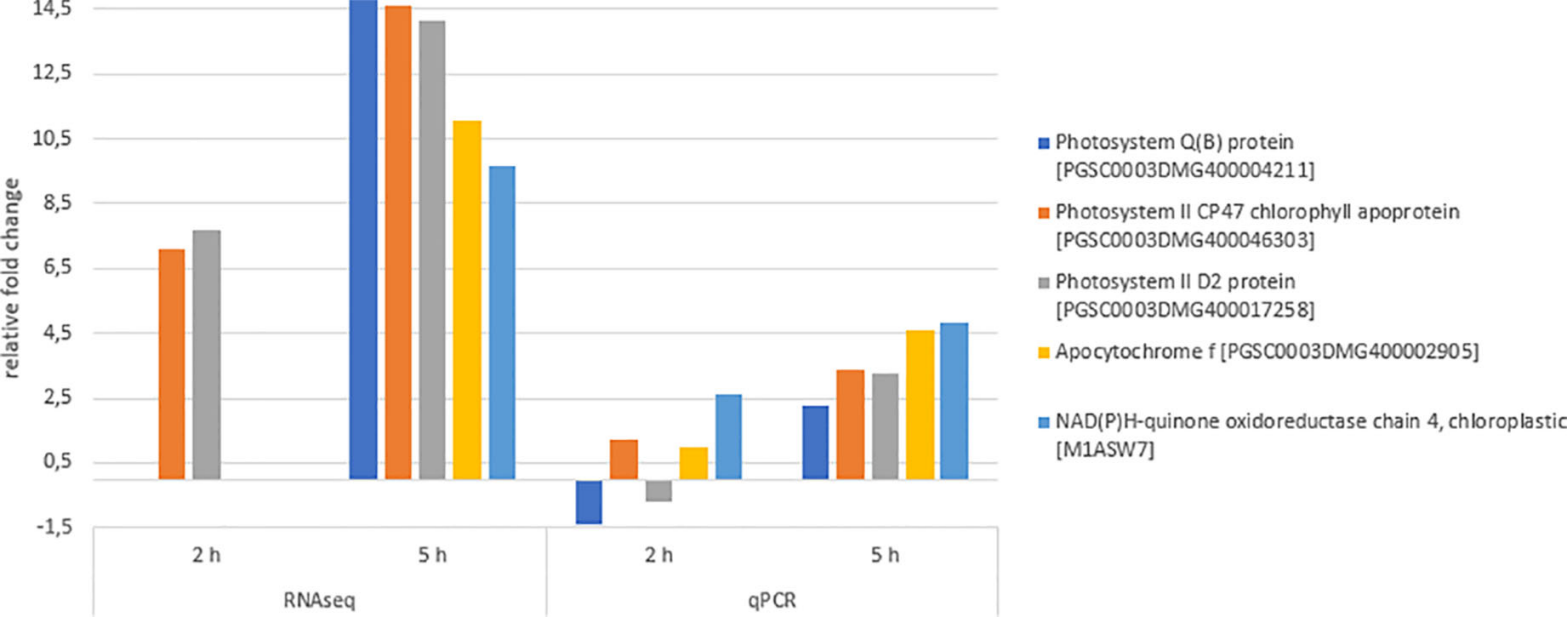
Differentially expressed genes (DEGs) validation *via* quantitative PCR (qPCR). Expression fold change of qPCR values was calculated using the efficiency corrected calculation model described by (Pfaffl, 2001). Blue bars indicate fold change 2 h after treatment, orange bars indicate fold change 5 h after treatment. Given expression changes of qPCR values are relative to water treatment. The Pearson correlation coefficient of both RNAseq and qPCR values 5 h after chitosan treatment was determined as 0.76, indicating a strong positive correlation.

qPCR experiments were performed to validate the gene expression results obtained by RNA sequencing (**Figure 8**). The qPCR results were in agreement with the sequencing results, showing the similar relative expression increases from 2 h to 5 h after chitosan treatment. The Pearson correlation coefficient between RNAseq and qPCR data for the genes quantified 5 h after treatment was determined as 0.76, indicating a strong positive correlation.

### Functional Analysis of Chitosan-Induced Reactions in Potato Leaves

**Figure 9:**
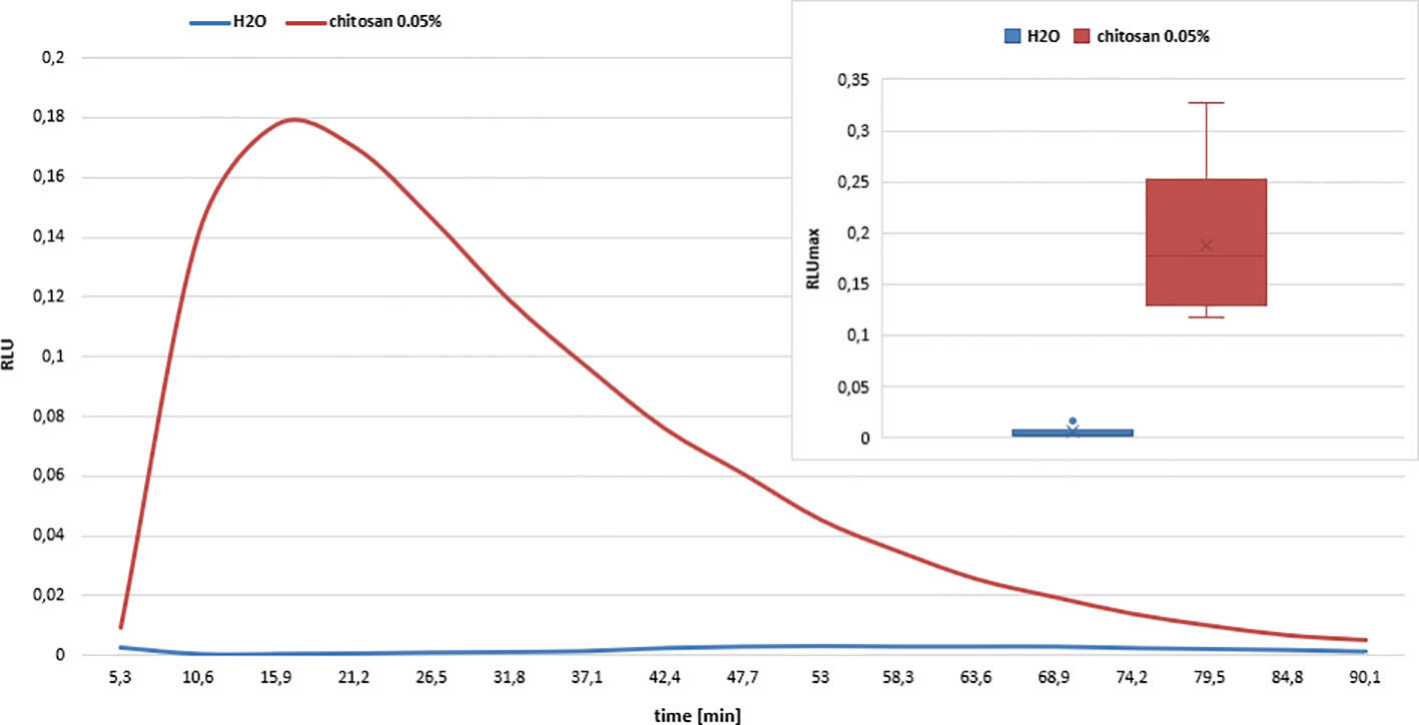
Oxidative burst response of chitosan treated potato leaf disks. Chitosan (FA 0.2, DPw 515, Đ_DP_ 2, 0.05%) elicits an oxidative burst reaction in potato leaf disks, as indicated by the rapid increase of relative light units (RLU) caused by H_2_O_2_ reaction with the luminol derivative L-012. Water-treatment of potato leaf disks did not result in any response. Reaction curves display mean values out of six technical replicates. Standard deviations are not shown to improve clarity. Box plots in the insert show the maximum RLU (RLUmax) values of chitosan- and water-treated potato leaves. The boxes contain 50% of all values with the median value given in a horizontal line and the mean vale given as a cross. The whiskers above and below the box represent data below the first or above the third quartile. Outliers are indicated as dots. The difference in response was significant according to one-way ANOVA analysis and *post hoc* Tukey test.

The common theme during gene induction in chitosan-treated potato leaves appears to be a correlation with electron transport. As electron transfer from NAD(P)H to oxygen in a rapid and transient oxidative burst is a known central, orchestrating event in the elicitation of disease resistance reactions, we quantified the H_2_O_2_ production in potato leaf disks after chitosan treatment, using the identical experimental setup as in the RNAseq experiment (**Figure 9**). We observed a typical oxidative burst reaction, with a fast onset, reaching a maximum at around 20 min and ending after around 90 min. Water treatment as a control did not result in an oxidative burst.

## Discussion

Chitosan treatments are long known to have the potential to protect plants, including potato, from disease, both owing to their direct antimicrobial activities and to the induction of the plant’s own defense systems. As a consequence, we had expected to predominantly observe chitosan-induced upregulation of defense-related genes, e.g., coding for pathogenesis related proteins or enzymes involved in the biosynthesis of phytoalexins and other secondary metabolites. However, this is not what we found. Instead, we observed a rather specific upregulation of genes involved in electron transport chains of photosynthesis and, to a lesser extent, of respiration. While a positive influence of chitosan treatments on plant growth and development is also well established, most studies investigating the effect of chitosan on plant productivity focus on time points days and weeks after treatment [as, e.g., reviewed in (Malerba and Cerana, 2016)]. As in our study, we targeted the earliest plant responses to chitosan treatment, this influence on photosynthetic electron transport and energy metabolism was unexpected. We hence suggest that the here observed gene activation displays an initial spark for all further observations.

The strong upregulation of genes coding for proteins of every complex of the photosynthetic light reaction clearly points to an activation of photosynthesis and hence, energy production upon chitosan treatment. Concerning PSII as the first protein complex, we observed upregulation of both *psbA* and *psbD*, coding for D1 and D2, the two main subunits of the reactive center core of PSII (Marder et al., 1987). Enhanced gene expression of *psbA* and *psbD* was accompanied by upregulation of *psbB*, encoding the PSII chlorophyll-binding protein CP47, which forms the inner light-harvesting complex of PSII together with the *psbC*-product CP43 (Barber et al., 1997; Bricker and Frankel, 2002). As PSII is the strongest known natural oxidizing agent (Vrettos and Brudvig, 2002), it suffers from oxidative damage and therefore displays shorter lifetimes in comparison to other photosynthetic compounds, necessitating a particularly high turnover rate (Yao et al., 2012). Thus, increased photosynthetic activity will have to be accompanied by increased biosynthesis of the oxidatively damaged proteins. However, upregulation of PSII most likely not only serves protein replacement, but is also part of an overall increase in photosynthetic capacity. This conclusion is supported by the fact that apart from the photosystems, genes coding for proteins related to both the cytochrome b_6_f complex and ATP synthase were upregulated. We observed significant upregulation of *petA* and *petB*, encoding the large subunits cytochrome f and cytochrome b_6_ of the complex, which indicates an increased electron flux and, thus, and increased capacity for photosynthetic activity. It is known that cytochrome b_6_f complex activity is closely linked to ATP synthase activity to ensure proton balance between stroma and lumen (Schöttler and Tóth, 2014). Accordingly, we observed upregulation of different subunits of the ATP synthase, namely genes encoding the α-subunits (*atpA*) as well as one gene each encoding a β-subunit (*atpB*) and a ϵ-subunit (*atpE*). All these upregulated genes encode proteins from the catalytic F_1_ head of the chloroplast ATP synthase, where α and β-subunits form the rotating α_3_β_3_-subcomplex responsible for ATP formation (Hahn et al., 2018). coregulation of the cytochrome b_6_f complex and the ATP synthase might provide evidence for a crosstalk between these complexes in potato, as generally assumed for higher plants (Schöttler and Tóth, 2014). In addition to linear electron transport, also cyclic electron transport appears to be enhanced, as indicated by upregulation of genes related to the type I NADPH dehydrogenase (NDH) complex responsible for cyclic electron transfer between PSI and the cytochrome b_6_f complex (Burrows et al., 1998). It has been shown that cyclic electron transfer *via* the NDH complex plays an important role in both C_3_ (Joet et al., 2002) and C_4_ (Ishikawa et al., 2016) plants to satisfy increased ATP demands. Increased NDH complex activity has been linked to the mitigation of heat and light stress (Essemine et al., 2017), oxidative damage, and other stresses (Yamori and Shikanai, 2016). Overall, the changes in photosynthetic energy metabolism induced by chitosan treatment, thus, most likely contribute to increased disease resistance and abiotic stress tolerance.

Activation of photosynthesis in response to chitosan treatment fits well to other findings. As an example, chitosan treatment led to overexpression of photosynthesis-related genes in strawberry fruits (Landi et al., 2017), increasing the fruit yield by more than 40% (Akter Mukta et al., 2017). Similarly, chitosan treatment of rice plants resulted in increased photosynthesis rates and higher biomass (Phothi and Theerakarunwong, 2017). Both examples suggest that chitosan treatment not only triggers the expression of photosynthesis-related genes, but indeed leads to increased photosynthetic activity. Accordingly, proteomic analysis of chitosan-treated rice plants showed significant upregulation of proteins involved in photosynthesis, carbohydrate metabolism and cell redox homeostasis (Chamnanmanoontham et al., 2014). As this protein expression was observed 24 h after chitosan treatment, these findings are in agreement with our observations of an upregulation of the corresponding genes in the first hours after chitosan treatment. Additionally, field trials with chitosan-treated potato plants resulted in up to 30% enhanced potato yields (Falcón-Rodríguez et al., 2017). Equally, foliar application of chitosan enhanced both growth and drought tolerance of potato plants (Muley et al., 2019). Based on our findings, this yield increase might be assigned to an activation of photosynthesis and resultant increased biomass production. Interestingly, potato virus Y infected potato plants show gene induction of photosynthesis-related processes including the light reaction within the first 12 h (Baebler et al., 2009) or 24 h (Stare et al., 2015) after inoculation. We therefore assume that chitosan treatment successfully mimics pathogen infection in potato, leading to a comparable response.

In our study, chitosan treatment induced genes coding for proteins involved in the electron transport chains of both the photosynthetic light reaction in the chloroplasts and the respiratory chain in the mitochondria. We observed upregulation of genes allocated to all protein complexes of the mitochondrial respiratory chain except for complex III. The respiratory electron transfer in plant mitochondria is not only important for energy supply *via* ATP synthesis, but can respond to different metabolic states of plant cells if altered due to environmental changes (Schertl and Braun, 2014). It is also known that not only the electron chain of the light reaction, but also the mitochondrial electron chain can react to light stress, e.g., supporting the chloroplasts to deal with excess NADPH (Yoshida et al., 2011). As several studies investigated the participation of mitochondria in producing ROS to regulate plant stress (Møller, 2001; Gleason et al., 2011; Huang S. et al., 2016), expression of mitochondrial respiratory chain-related genes might also be related to defense responses, as discussed below.

Increased activities of the photosynthetic and respiratory electron transfer chains will invariably lead to increased ROS production. In addition, both organelles are able to deliberately generate ROS as signal molecules (Foyer and Noctor, 2003) or antimicrobial agents (Choudhury et al., 2017), possibly even mediating and amplifying ROS signals deriving from the apoplast (Joo, 2005). While ROS concentrations are usually kept low by the action of detoxifying antioxidant systems, stress conditions can lead to retrograde ROS signaling (Navrot et al., 2007), e.g., between chloroplast and nucleus in the event of high light conditions (Galvez-Valdivieso and Mullineaux, 2010). In total, the formation of ROS in different cellular compartments in response to different conditions and their export into the cytosol establish extensive crosstalk of ROS in plant cells, integrating a broad range of cellular processes including gene expression, primary and secondary metabolism, and direct protection against diseases (Frederickson Matika and Loake, 2014). By regulating cellular redox homeostasis, ROS crosstalk ultimately provides information to the plant on the current energy status for growth and general development (Foyer and Noctor, 2009). Chitosan is well known to elicit an apoplastic oxidative burst in many plant species (Malerba and Cerana, 2016; Lopez-Moya et al., 2019). Recent *in vivo* quantification of intracellular H_2_O_2_ in *Arabidopsis* revealed that apoplastic ROS can enter the cytosol and the mitochondrial matrix to modulate cell signals (Nietzel et al., 2018). Chitosan-triggered increased activities of organellar electron transport chains may well contribute to this complex ROS signaling.

It is furthermore well known that chitosan treatment can act as plant priming, enabling a faster and more efficient response to upcoming biotic or abiotic challenges (Frost et al., 2008; Mauch-Mani et al., 2017), and chitosan has recently been shown to induce priming in rice cells (Basa et al., 2020). Priming agents are thought to act on redox signaling, altering the overall oxidative environment of plant cells which eventually puts plants in an alarm state (González-Bosch, 2018). Our study suggests that chitosan-induced priming activity of chitosan may involve activation of redox-sensitive genes to support primary metabolism and defense preparation against prospective stress situation.

Upregulation of genes related to ROS formation in plant organelles in response to chitosan treatment may suggest prolonged ROS accumulation, not only for signaling and priming, but also directly serving disease resistance, as also suggested by chitosan-triggered defense-related gene expression. Particularly, we observed the early upregulation of a LRR-RLK and of one type of WRKY transcription factor. LRR-RLKs play central roles in signaling during pathogen perception (Afzal et al., 2008), while WRKY transcription factors constitute a major transcription factor family in plants, regulating a broad range of processes including biotic and abiotic stresses (Phukan et al., 2016). The here observed LRR-RLK was for instance also found to be upregulated in the wild potato *Solanum commersonii* upon *Ralstonia solanacearum* infection (Zuluaga et al., 2015), and LRR-RLKs are also known to be involved in ROS signaling (Eckardt, 2017). As no chitosan specific receptor has been described in plants so far, it is tempting to speculate that this LRR-RLK is involved in chitosan perception, possibly triggering a signal cascade involving mitogen-activated protein (MAP) kinases as described for chitin perception in rice and *Arabidopsis* plants (Kawasaki et al., 2017). Based on a classification by (Huang and Liu, 2013), the WRKY transcription factor found in this study is classified as StWRKY22, located on chromosome 3 and grouped into group III, the group that contains WRKY transcription factors influencing disease resistance (Wang et al., 2015; Huang Y. et al., 2016). Upregulation of both LRR-RLK and StWRKY22 clearly indicates that chitosan triggers defense responses in potato leaves within the first few hours after treatment. In addition, we observed upregulation of a lipoxygenase, an enzyme involved in the synthesis of many signaling compounds (Porta and Rocha-Sosa, 2002) and known to be induced during pathogen defense (Ocampo et al., 1986). The octadecanoid pathway in which lipoxygenase catalyzes the first step, results in the production of jasmonic acid (JA), an important signaling molecule mediating plant responses toward both biotic and abiotic stresses (Ruan et al., 2019). Chitosan -induced upregulation of lipoxygenase, thus, is in agreement with the long known JA accumulation in response to chitosan treatment (Doares et al., 2006; Kim et al., 2014).

Further evidence for a connection between upregulation of ROS-related genes and disease resistance and also for the already mentioned redox-sensitive priming activity of chitosan is provided by the observation of an oxidative burst response in potato leaf disks upon treatment with chitosan. Chitosan is well known to display eliciting activity in plants (Malerba and Cerana, 2016), possibly perceived *via* chitin receptors (Kaku et al., 2006; Gubaeva et al., 2018). Downstream signaling leading to the activation of plant immunity involves the generation of ROS, activation of phytohormone crosstalk, the production of pathogenesis-related proteins as well as other responses involved in warding off pathogens (Jones and Dangl, 2006). However, chitosans differ in their structural parameters such as their degree of polymerization and degree of acetylation, and both parameters strongly influence the biological activities of chitosans (Vander et al., 1998; Cord-Landwehr et al., 2020; Wattjes et al., 2020). Also, different plant species may react differently to the same chitosan (Santos et al., 2008). Therefore, it was important to show that the chitosan used for the transcriptomic study indeed induces a disease resistance response in the potato genotype used. As a central orchestrating event in the induction of resistance reactions and based on the observation of massive upregulation of genes encoding components of electron transport chains, we chose elicitation of the oxidative burst as a read-out in this functional verification experiment. The observed rapid and early ROS release *via* oxidative burst reactions in response to chitosan might explain the subsequent upregulation of ROS-related genes, as proteins involved in ROS signaling are vulnerable to damage *via* ROS scavenging or forwarding in signaling processes (Sharma et al., 2012). Hence, the observed upregulation of genes involved in ROS crosstalk might provide evidence for both a general armament of such structures and a replacement of already exhausted structures through the observed, preceding oxidative burst reactions, providing new insights for a functional connection between nucleus-dependent and nucleus-independent signaling pathways of chitosan triggered ROS reactions (Schmitt et al., 2014). Clearly, the here described transcriptomic approach should be complemented in future by a metabolomic study in order to gain a more comprehensive understanding of chitosan-triggered immunity. In addition, observation of both upregulation of ROS-related genes, eventually supporting photosynthesis, and the oxidative burst itself provides evidence for a positive feedback of primary metabolism and defense, as sugar concentrations are known to regulate ROS generation and removal (Couée et al., 2006; Keunen et al., 2013). Furthermore, ROS are known to influence plant growth as they tend to accumulate in meristems (Huang et al., 2019).In this study, surprisingly few genes were significantly overexpressed in comparison to transcriptomic approaches of chitosan-treated strawberry (Landi et al., 2017) and avocado (Xoca-Orozco et al., 2017) fruits. While the treatment in this study was based on a well-characterized, pure chitosan that has been optimized for plant disease protection, a standard commercial chitosan sourced from Sigma-Aldrich was used for the avocado treatment, and a chitosan-based commercial product from ChiPro GmbH for strawberry treatment. Also, to avoid nonspecific stress reactions, we used our chitosan at a very low concentration (0.05%) which we know to give optimal plant protection, while much higher concentrations were used in the other studies (1.5% in avocado, 1% in strawberry). Hence, we believe that one reason for the comparably high number of differential gene expression in the other studies might be the usage of less characterized or less pure chitosans at much higher concentrations, leading to nonspecific effects.

In summary, our study demonstrates that chitosan perception leads to an activation of primary metabolism and thus, indirectly, plant defense. The strongly dominating focus on electron chains in chloroplasts and mitochondria indicates increased energy production and intracellular crosstalk, ultimately resulting in a more productive metabolic state. These responses concomitantly contribute to the often described increased disease resistance and abiotic stress tolerance as well as promotion of plant growth and development upon chitosan treatment, leading to higher productivity of crop plants upon chitosan treatment.

## Data Availability Statement

The datasets presented in this study can be found in online repositories. The names of the repository/repositories and accession number(s) can be found below: https://www.ebi.ac.uk/ena/browser, PRJEB36930.

## Author Contributions

BMM conceived the study and supervised the experiments. PL performed the experiments. RS supported the data analysis. BMM, PL and RS wrote the manuscript.

## Funding

This work was performed as part of the FunChi project (project ID 22032315), supported by the Fachagentur Nachwachsende Rohstoffe (FNR) of the German Federal Ministry of Food and Agriculture (BMEL) in the framework of the European Comission's FP7-KBBE program ERA-IB-15-08.

## Conflict of Interest

The authors declare that the research was conducted in the absence of any commercial or financial relationships that could be construed as a potential conflict of interest.
