# Supplementary figures and images for: Transcriptome Analysis of Solanum Tuberosum Genotype RH89-039-16 in Response to Chitosan
Source: Front Plant Sci. 2020 Aug 5;11:1193. doi: 10.3389/fpls.2020.01193 (PMC7438930; doi:10.3389/fpls.2020.01193)

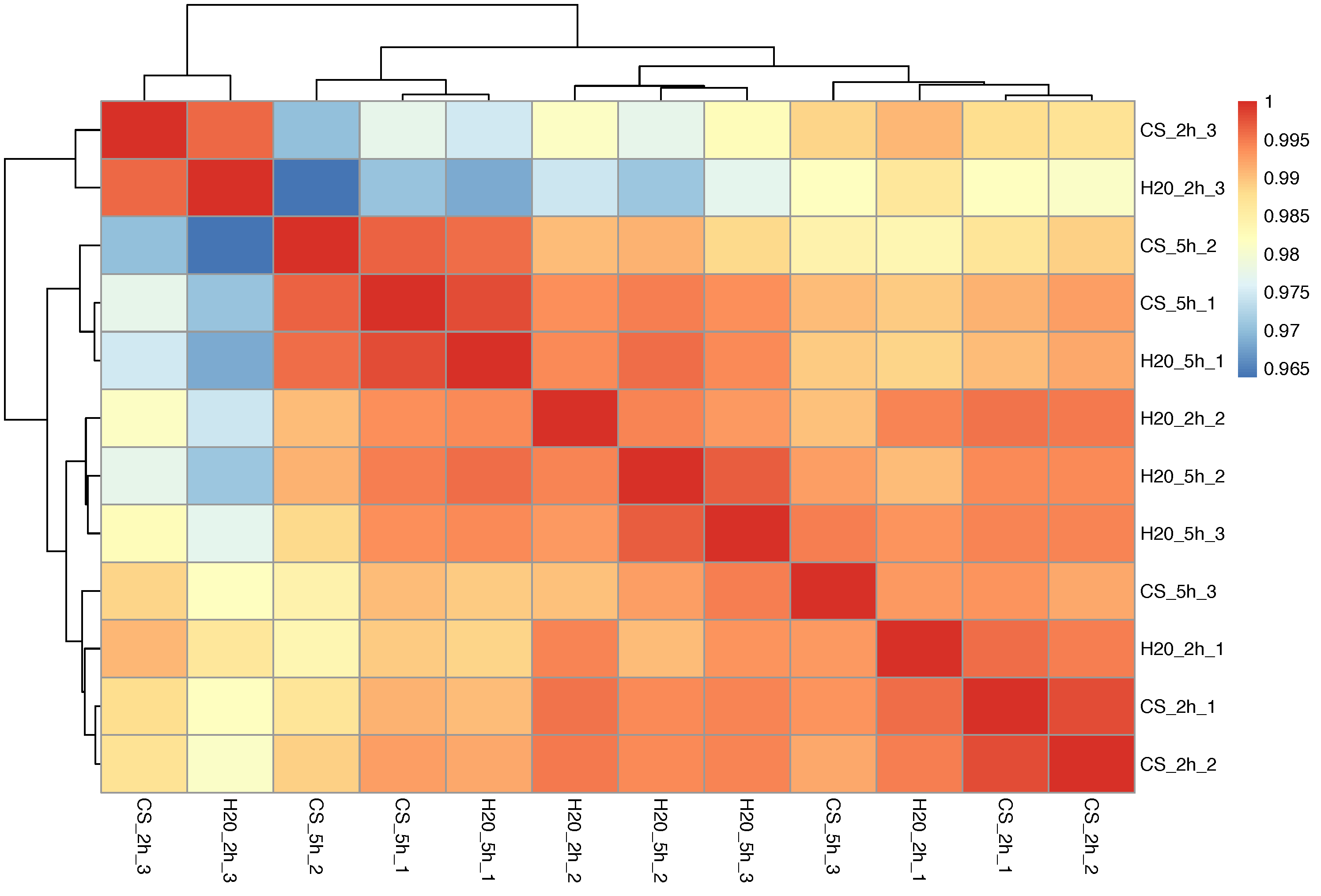

Supplement: Supplementary file 1 [file Image_1.tif]

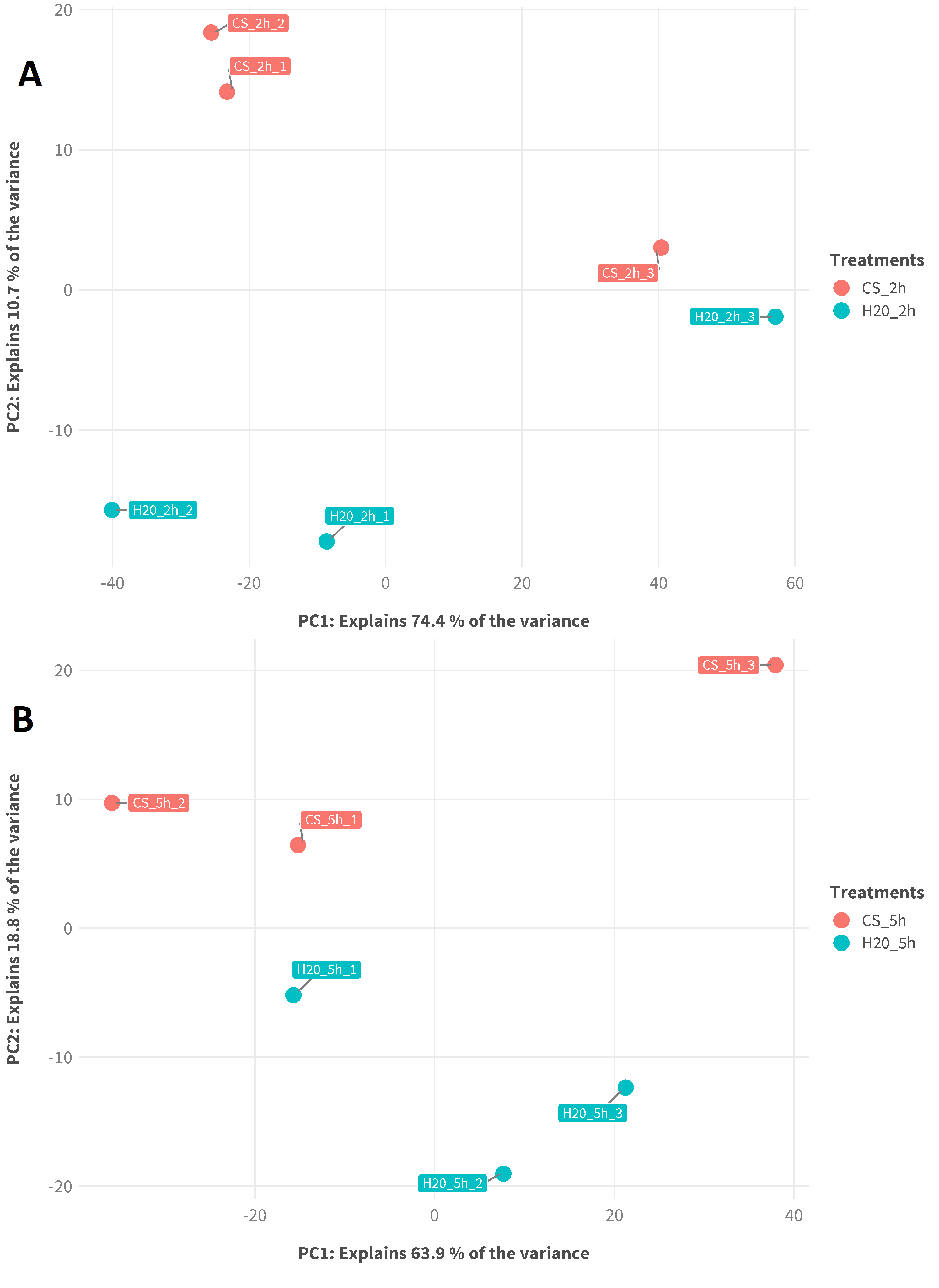

Supplement: Supplementary file 2 [file Image_2.tif]

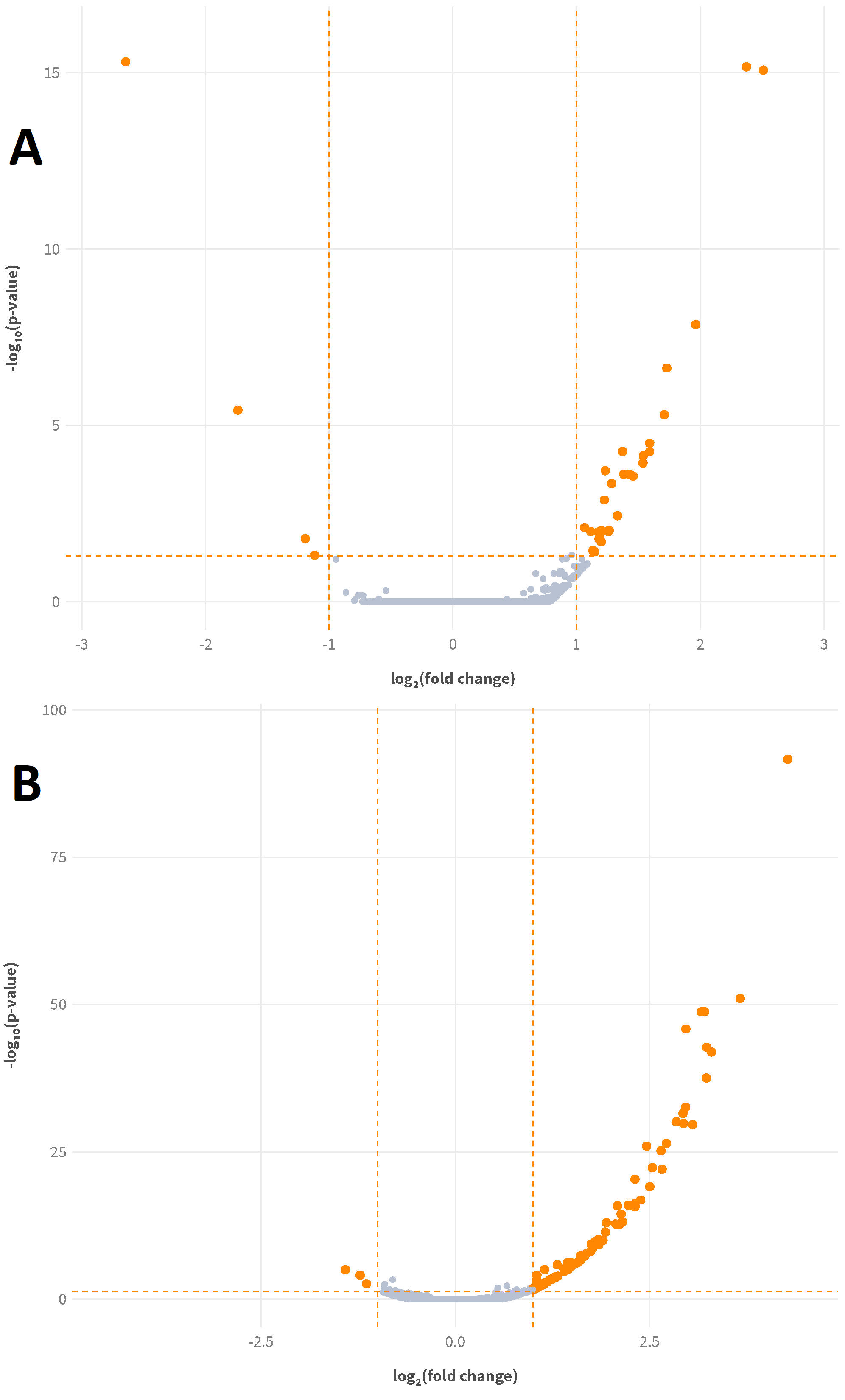

Supplement: Supplementary file 3 [file Image_3.tif]

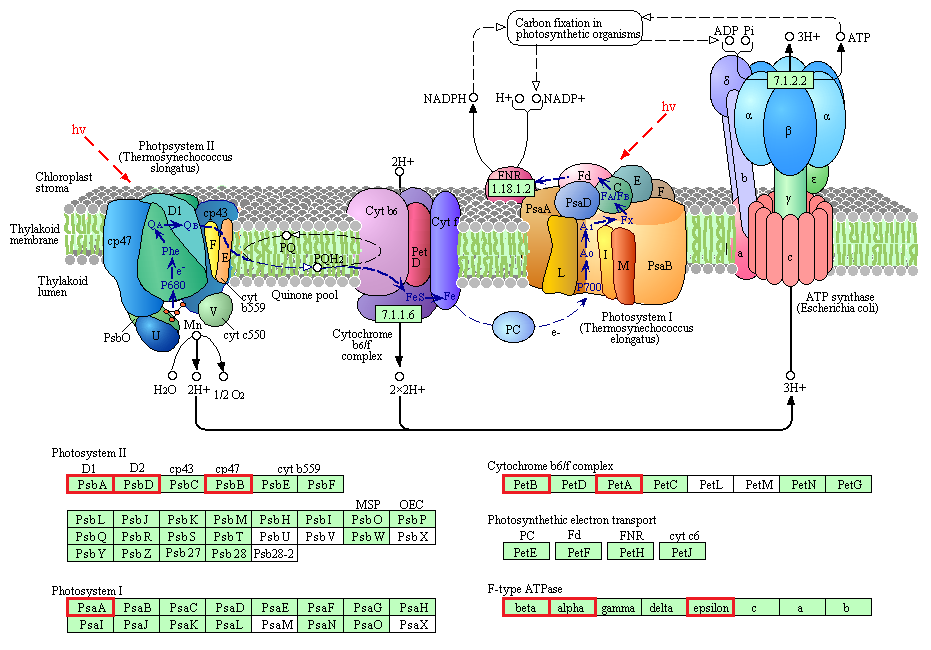

Supplement: Supplementary file 4 [file Image_4.tif]
