# Supplementary material for: Transcriptome Analysis of Solanum Tuberosum Genotype RH89-039-16 in Response to Chitosan
Source: Front Plant Sci. 2020 Aug 5;11:1193. doi: 10.3389/fpls.2020.01193 (PMC7438930; doi:10.3389/fpls.2020.01193)
Supplement: Supplementary file 6 [file Table_2.docx]

Supplementary Figure Captions

Supplementary Figure 1: Heatmap presenting Pearson’s correlation coefficient calculated pair-wise for all samples. The data is regularized log_2_-transformed and clustered by rows and columns.

**Supplementary** **Figure 2:** **Principal component analysis (PCA) of time point 2 h (A) and time point 5 h (B) samples.** The data is regularized log_2_-transformed.

Supplementary Figure 3: Volcano plots of DEGs 2 h (A) and DEGs 5 h (B) after chitosan treatment. The significantly DEGs with adjusted p <0.05 and absolute log2 fold change >1 are shown in orange, non-significantly DEGs are shown in grey.

Supplementary Figure 4: KEGG pathway map (entry sot00195) of photosynthetic light reaction in potato. Up-regulated genes are marked in red boxes. Chloroplast-encoded genes are indicated in green, white-colored boxes contain nucleus-encoded genes. Permission to use this KEGG pathway map (entry sot00195) was granted (Ogata et al. 1999; Kanehisa et al. 2017; 2019).
