# Supplementary material for: Transcriptome Analysis of Solanum Tuberosum Genotype RH89-039-16 in Response to Chitosan
Source: Front Plant Sci. 2020 Aug 5;11:1193. doi: 10.3389/fpls.2020.01193 (PMC7438930; doi:10.3389/fpls.2020.01193)
Supplement: Supplementary file 7 [file Table_3.docx]

Supplementary Tables

Supplementary Table 1: Alignment statistics of the samples showing total number of reads in samples, average length of mapped reads, and percentages of reads mapping to unique positions, reads mapping to multiple positions, and reads that could not be mapped to the reference.

| **Sample name** | **Number of reads** | **Read length [bp]** | **Uniquely mapped [%]** | **Multi mapped [%]** | **Unmapped [%]** |
| --- | --- | --- | --- | --- | --- |
| CS_2h_1 | 32873578 | 50 | 85.72 | 13.00 | 1.27 |
| CS_2h_2 | 27720381 | 50 | 83.65 | 14.62 | 1.73 |
| CS_2h_3 | 29860107 | 50 | 82.76 | 15.71 | 1.53 |
| CS_5h_1 | 32222315 | 50 | 71.04 | 27.77 | 1.19 |
| CS_5h_2 | 35356577 | 50 | 67.11 | 31.57 | 1.31 |
| CS_5h_3 | 46735394 | 50 | 64.37 | 34.49 | 1.13 |
| H_2_O_2h_1 | 39437564 | 49 | 91.12 | 7.24 | 1.63 |
| H_2_O_2h_2 | 30732963 | 49 | 91.04 | 7.38 | 1.57 |
| H_2_O_2h_3 | 38570413 | 49 | 90.50 | 7.80 | 1.69 |
| H_2_O_5h_1 | 39756101 | 50 | 90.28 | 8,15 | 1.56 |
| H_2_O_5h_2 | 29850088 | 50 | 90.45 | 7.92 | 1.64 |
| H_2_O_5h_3 | 29892271 | 50 | 90.19 | 7.04 | 1.77 |

| **Annotation** | **Gene** | **ID** | **Primer sequences** | **Amplicon size** | **PCR efficiency** | **R^2^** |
| --- | --- | --- | --- | --- | --- | --- |
| Elongation factor 1-alpha | ef1-α | PGSC0003DMG400023270 | fwd TGAGGCAAACTGTTGCTGTC  rev TGGAAACACCAGCATCACAC | 126 bp | 102,8 % | 0,97 |
| *S. tuberosum* gene for 18S rRNA | 18S rRNA | GenBank: X67238.1 | fwd TGATAACTCGACGGATCGCA  rev TGGATGTGGTAGCCGTTTCT | 166 bp | 87,7 % | 0,99 |
|  | | | | | | |
| Photosystem Q(B) protein | psbA | PGSC0003DMG400004211 | fwd TAGAGAGACGCGAAAGCGAA  rev TCTACTGGAGGAGCAGCAATG | 163 bp | 72,3 % | 0,99 |
| Photosystem II CP47 chlorophyll apoprotein | psbB | PGSC0003DMG400046303 | fwd TCGAAGAGTTAGTGCTGGGC  rev TGTCCTAACCATCCAACCGC | 174 bp | 108,1 % | 0,99 |
| Photosystem II D2 protein | psbD | PGSC0003DMG400017258 | fwd GTAGGGGGTTGGTTCACAGG  rev CTTGTGCTTCAGGACCCCAT | 163 bp | 112,8 % | 0,99 |
| Apocytochrome f | petA | PGSC0003DMG400002905 | fwd CCACAAGCGGTACTTCCTGA  rev AATACGATCGGGAGGGGCTA | 162 bp | 105,4 % | 0,98 |
| NAD(P)H-quinone oxidoreductase chain, chloroplastic | NDH | PGSC0003DMG401011339 | fwd AAACAATACGAGCCGCCAGA  rev TGTATTCGGCCTCGGAAACG | 133 bp | 103,5 % | 0,99 |

Supplementary Table 2: qPCR primers for DEG validation

Supplementary Table 3: Gene descriptions and corresponding transcriptomic gene IDs of described and discussed DEGs. Downregulated genes are indicated in red.

| **DEGs 2 h after treatment only** | |
| --- | --- |
| **Gene ID** | **Gene description** |
| PGSC0003DMG400017713 | LRR receptor-like serine/threonine-protein kinase |
| PGSC0003DMG400017713 | WRKY transcription factor |
| PGSC0003DMG400009103 | Pollen Ole e 1 allergen and extensin |
| PGSC0003DMG400001380 | Proline-rich cell wall protein |
| PGSC0003DMG400009783 | PRA1 family protein F3 |
| PGSC0003DMG401031172 | Leucoanthocyanidin dioxygenase |
| PGSC0003DMG400027333 | Peroxisomal 3-hydroxyisobutyryl-coenzyme A hydrolase |

| **DEGs 5 h after treatment only** | |
| --- | --- |
| **Gene ID** | **Gene description** |
| PGSC0003DMG401011339 | NAD(P)H-quinone oxidoreductase chain 4, chloroplastic |
| PGSC0003DMG401011339 | NAD(P)H-quinone oxidoreductase subunit 1, chloroplastic |
| PGSC0003DMG402008783 | NAD(P)H-quinone oxidoreductase subunit K, chloroplastic |
| PGSC0003DMG400006986 | NAD(P)H-quinone oxidoreductase subunit 3, chloroplastic |
| PGSC0003DMG400015304 | ATP synthase subunit alpha, mitochondrial (*atpA*) |
| PGSC0003DMG401022238 | ATP synthase epsilon chain, chloroplastic (*atpE*) |
| PGSC0003DMG400025106 | ATP synthase subunit alpha, mitochondrial (*atpA*) |
| PGSC0003DMG400013849 | ATP synthase subunit alpha, chloroplastic (*atpA*) |
| PGSC0003DMG400008476 | H^+^-transporting two-sector ATPase beta chain (*atpB*) |
| PGSC0003DMG400034122 | Photosystem Q(B) protein (*psbA*) |
| PGSC0003DMG400004211 | Apocytochrome f (*petA*) |
| PGSC0003DMG400002905 | NADH-ubiquinone oxidoreductase 49 kDa subunit |
| PGSC0003DMG400030943 | NADH-ubiquinone oxidoreductase chain 2 |
| PGSC0003DMG400003375 | NADH-ubiquinone oxidoreductase 49 kDa subunit |
| PGSC0003DMG400013204 | NADH-ubiquinone oxidoreductase chain 2 |
| PGSC0003DMG400013204 | NADH dehydrogenase |
| PGSC0003DMG400020931 | NtEIG-E80 protein |
| PGSC0003DMG400021388 | PAR-1c protein |
| PGSC0003DMG400011087 | Orf122b protein |
| PGSC0003DMG400014347 | 9-cis-epoxycarotenoid dioxygenase |
| PGSC0003DMG400041193 | Glutaredoxin family protein |
| PGSC0003DMG400004311 | Alpha-DOX2 |
| PGSC0003DMG400008952 | Ethylene-responsive transcription factor |

| DEGs both 2 h and 5 h after treatment | |
| --- | --- |
| **Gene ID** | **Gene description** |
| PGSC0003DMG400012033 | Photosystem I P700 chlorophyll a apoprotein (*psbA*) |
| PGSC0003DMG400015960 | Photosystem I P700 chlorophyll a apoprotein A1 (*psbA)* |
| PGSC0003DMG400005372 | Photosystem I P700 chlorophyll a apoprotein A1 (*psbA)* |
| PGSC0003DMG400017258 | Photosystem II D2 protein (*psbD*) |
| PGSC0003DMG400046303 | Photosystem II CP47 chlorophyll apoprotein (*psbB*) |
| PGSC0003DMG400019419 | Cytochrome b6 (*petB*) |
| PGSC0003DMG400033037 | Ribulose 1,5-bisphosphate carboxylase/oxygenase large subunit (*rbcL*) |

Supplementary Table 4: Enrichment analysis of DEGs 2 h after chitosan treatment. All listed GO terms were significantly enriched with adjusted p-values below 0.05.

| **GO ID** | **GO term description** | **Gene ratio** | **Background ratio** | **Associated genes** |
| --- | --- | --- | --- | --- |
| GO:0009579 | thylakoid | 3/16 | 3/13656 | PGSC0003DMG400012033 PGSC0003DMG400015960 PGSC0003DMG400005372 |
| GO:0009522 | photosystem I | 3/16 | 7/13656 | PGSC0003DMG400012033 PGSC0003DMG400015960 PGSC0003DMG400005372 |
| GO:0015979 | photosynthesis | 3/16 | 45/13656 | PGSC0003DMG400012033 PGSC0003DMG400015960 PGSC0003DMG400005372 |
| GO:0019684 | photosynthesis,  light reaction | 2/16 | 15/13656 | PGSC0003DMG400017258 PGSC0003DMG400046303 |
| GO:0016021 | integral component of membrane | 4/16 | 460/13656 | PGSC0003DMG400012033 PGSC0003DMG400015960 PGSC0003DMG400005372 PGSC0003DMG400002971 |
| GO:0009055 | electron carrier activity | 2/16 | 163/13656 | PGSC0003DMG400000620 PGSC0003DMG400002971 |

**Supplementary Table 5:** Enrichment analysis of DEGs 5 h after chitosan treatment. All listed GO terms were significantly enriched with adjusted p-values below 0.05.

| **GO ID** | **GO term description** | **Gene ratio** | **Background ratio** | **Associated genes** |
| --- | --- | --- | --- | --- |
| GO:0009579 | thylakoid | 3/42 | 3/13656 | PGSC0003DMG400005372 PGSC0003DMG400012033 PGSC0003DMG400015960 |
| GO:0009522 | photosystem I | 3/42 | 7/13656 | PGSC0003DMG400005372 PGSC0003DMG400012033 PGSC0003DMG400015960 |
| GO:0008137 | NADH dehydrogenase (ubiquinone) activity | 3/42 | 12/13656 | PGSC0003DMG401011339 PGSC0003DMG400021388 PGSC0003DMG400015304 |
| GO:0019684 | photosynthesis, light reaction | 3/42 | 15/13656 | PGSC0003DMG400004211 PGSC0003DMG400017258 PGSC0003DMG400046303 |
| GO:0015979 | photosynthesis | 4/42 | 45/13656 | PGSC0003DMG400005372 PGSC0003DMG400012033 PGSC0003DMG400002905 PGSC0003DMG400015960 |
| GO:0015986 | ATP synthesis coupled proton transport | 4/42 | 49/13656 | PGSC0003DMG400001014 PGSC0003DMG400025106 PGSC0003DMG400008476 PGSC0003DMG400026520 |
| GO:0046933 | proton-transporting ATP synthase activity, rotational mechanism | 3/42 | 22/13656 | PGSC0003DMG400001014 PGSC0003DMG400034122 PGSC0003DMG400025106 |
| GO:0046961 | proton-transporting ATPase activity, rotational mechanism | 3/42 | 22/13656 | PGSC0003DMG400001014 PGSC0003DMG400034122 PGSC0003DMG400025106 |
| GO:0009055 | electron carrier activity | 5/42 | 163/13656 | PGSC0003DMG400002905 PGSC0003DMG400002971 PGSC0003DMG400000621 PGSC0003DMG400000620 PGSC0003DMG400008952 |
| GO:0016820 | hydrolase activity, acting on acid anhydrides, catalyzing transmembrane movement of substances | 2/42 | 8/13656 | PGSC0003DMG400011350 PGSC0003DMG400008476 |
| GO:0042773 | ATP synthesis coupled electron transport | 2/42 | 8/13656 | PGSC0003DMG401011339 PGSC0003DMG400021388 |
| GO:0045156 | electron transporter, transferring electrons within the cyclic electron transport pathway of photosynthesis activity | 2/42 | 10/13656 | PGSC0003DMG400004211 PGSC0003DMG400017258 |
| GO:0045261 | proton-transporting ATP synthase complex, catalytic core F(1) | 2/42 | 17/13656 | PGSC0003DMG400001014 PGSC0003DMG400025106 |
| GO:0003735 | structural constituent of ribosome | 5/42 | 308/13656 | PGSC0003DMG400021744 PGSC0003DMG400003499 PGSC0003DMG400014076 PGSC0003DMG400033612 PGSC0003DMG401008792 |
| GO:0006412 | translation | 5/42 | 346/13656 | PGSC0003DMG400021744 PGSC0003DMG400003499 PGSC0003DMG400014076 PGSC0003DMG400033612 PGSC0003DMG401008792 |
| GO:0003899 | DNA-directed RNA polymerase activity | 2/42 | 43/13656 | PGSC0003DMG402017663 PGSC0003DMG400014075 |
| GO:0016021 | integral component of membrane | 5/42 | 460/13656 | PGSC0003DMG400005372 PGSC0003DMG400012033 PGSC0003DMG400015960 PGSC0003DMG400011350 PGSC0003DMG400002971 |
| GO:0005840 | ribosome | 4/42 | 302/13656 | PGSC0003DMG400003499 PGSC0003DMG400014076 PGSC0003DMG400033612 PGSC0003DMG401008792 |
| GO:0055114 | oxidation-reduction process | 8/42 | 1141/13656 | PGSC0003DMG401011339 PGSC0003DMG400021388 PGSC0003DMG402008783 PGSC0003DMG400032207 PGSC0003DMG400030943 PGSC0003DMG402000506 PGSC0003DMG400015304 PGSC0003DMG400009822 |
| GO:0005507 | copper ion binding | 2/42 | 94/13656 | PGSC0003DMG400000621 PGSC0003DMG400000620 |
| GO:0005622 | intracellular | 4/42 | 552/13656 | PGSC0003DMG400003499 PGSC0003DMG400014076 PGSC0003DMG400033612 PGSC0003DMG401008792 |
| GO:0020037 | heme binding | 4/42 | 639/13656 | PGSC0003DMG400002905 PGSC0003DMG400002971 PGSC0003DMG402000506 PGSC0003DMG400009822 |
| GO:0016020 | membrane | 5/42 | 940/13656 | PGSC0003DMG400004211 PGSC0003DMG400019419 PGSC0003DMG400046303 PGSC0003DMG400016897 PGSC0003DMG402008783 |
| GO:0005506 | iron ion binding | 3/42 | 567/13656 | PGSC0003DMG400002905 PGSC0003DMG400002971 PGSC0003DMG40000982 |
